# (*E*)-*N*′-[1-(4-Amino­phen­yl)ethyl­idene]benzohydrazide

**DOI:** 10.1107/S1600536808019004

**Published:** 2008-06-28

**Authors:** Shang Shan, Yu-Liang Tian, Shan-Heng Wang, Wen-Long Wang, Ying-Li Xu

**Affiliations:** a College of Chemical Engineering and Materials Science, Zhejiang University of Technology, People’s Republic of China

## Abstract

Crystals of the title compound, C_15_H_15_N_3_O, were obtained from a condensation reaction of benzohydrazide and 1-(4-amino­phen­yl)ethanone. The mol­ecule assumes an *E* configuration with the amino­phenyl and benzohydrazide units located on opposite sites of the C=N double bond. In the crystal structure, the benzene rings of the mol­ecule are slightly twisted with respect to the central hydrazide, the dihedral angles being 18.22 (12) and 27.62 (12)°. The crystal structure contains inter­molecular N—H⋯O and weak C—H⋯N hydrogen bonding.

## Related literature

For general background, see: Okabe *et al.* (1993 ▶); Shan *et al.* (2003 ▶). For a related structure, see: Shan *et al.* (2008 ▶).
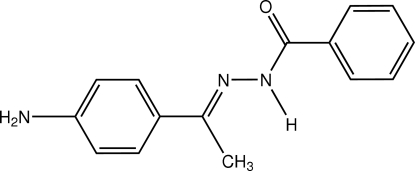


## Experimental

### 

#### Crystal data

C_15_H_15_N_3_O
*M* *_r_* = 253.30
Monoclinic, 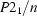

*a* = 12.261 (9) Å
*b* = 5.324 (4) Å
*c* = 19.882 (15) Å
β = 94.57 (2)°
*V* = 1293.7 (17) Å^3^
*Z* = 4
Mo *K*α radiation
μ = 0.08 mm^−1^
*T* = 295 (2) K
0.42 × 0.36 × 0.32 mm

#### Data collection

Rigaku R-AXIS RAPID IP diffractometer
Absorption correction: none
10914 measured reflections
2303 independent reflections
1594 reflections with *I* > 2σ(*I*)
*R*_int_ = 0.042

#### Refinement

*R*[*F* ^2^ > 2σ(*F* ^2^)] = 0.048
*wR*(*F* ^2^) = 0.156
*S* = 1.05
2303 reflections
174 parameters
H-atom parameters constrained
Δρ_max_ = 0.21 e Å^−3^
Δρ_min_ = −0.15 e Å^−3^

### 

Data collection: *PROCESS-AUTO* (Rigaku, 1998 ▶); cell refinement: *PROCESS-AUTO*; data reduction: *CrystalStructure* (Rigaku/MSC, 2002 ▶); program(s) used to solve structure: *SIR92* (Altomare *et al.*, 1993 ▶); program(s) used to refine structure: *SHELXL97* (Sheldrick, 2008 ▶); molecular graphics: *ORTEP-3 for Windows* (Farrugia, 1997 ▶); software used to prepare material for publication: *WinGX* (Farrugia, 1999 ▶).

## Supplementary Material

Crystal structure: contains datablocks I, global. DOI: 10.1107/S1600536808019004/xu2432sup1.cif

Structure factors: contains datablocks I. DOI: 10.1107/S1600536808019004/xu2432Isup2.hkl

Additional supplementary materials:  crystallographic information; 3D view; checkCIF report

## Figures and Tables

**Table 1:** Hydrogen-bond geometry (Å, °)

*D*—H⋯*A*	*D*—H	H⋯*A*	*D*⋯*A*	*D*—H⋯*A*
N3—H3*B*⋯O1^i^	0.86	2.44	3.169 (3)	143
C15—H15*C*⋯N2^ii^	0.96	2.62	3.468 (3)	147

Symmetry codes: (i) 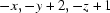; (ii) 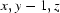.

**Fig. 1.**
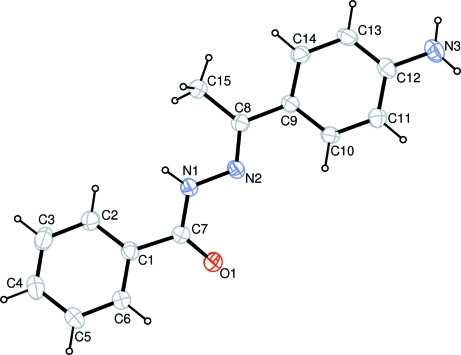
The molecular structure of the title compound with 50% probability displacement ellipsoids for non-H atoms.

## supplementary crystallographic information

### Comment

Since some hydrazone derivatives have shown to be potential DNA damaging and mutagenic agents (Okabe *et al.*, 1993), a series of new hydrazone derivatives have been prepared in our laboratory (Shan *et al.*, 2003). As part of the ongoing investigation, the title compound has recently been prepared and its crystal structure is reported here.

The molecular structure of the title compound is shown in Fig. 1. The N2—C8 bond distance of 1.292 (2) Å indicates a typical C═N double bond. The aminophenyl and benzohydrazide moieties located on the opposite sites of the C═N bond, the molecule assumes an E configuration, similat to that found in a related compound, (*E*)-acetylpyrazine 4-nitrophenylhydrazone (Shan *et al.*, 2008). The terminal benzene rings are slightly twisted to the central hydrazide (O1/C7/N1/N2), with dihedral angles of 18.22 (12)° between C1-benzene and hydrazide planes and 27.62 (12)° between aminophenylethylidene and hydrazide planes, indicating the approximately co-planar molecular structure except for methyl H atoms.

The crystal structure contains molecular classic N—H···O hydrogen bonding and weak C—H···N hydrogen bonding (Table 1).

### Experimental

Benzohydrazide (0.27 g, 2 mmol) was dissolved in ethanol (10 ml), then acetic acid (0.1 ml) was added to the ethanol solution with stirring. The solution was heated at 333 K for several minutes until the solution cleared. 1-(4-aminophenyl)ethanone (0.27 g, 2 mmol) was then added slowly into the solution, and the mixture was kept at 333 K with continuous stirring for 6 h. After the solution had cooled to room temperature yellow powder crystals appeared. The powder crystals were separated and washed with water three times. Recrystallization from an absolute ethanol yielded well shaped single crystals of the title compound.

### Refinement

Methyl H atoms were placed in calculated positions with C—H = 0.96 Å and the torsion angle was refined to fit the electron density, *U*_iso_(H) = 1.5*U*_eq_(C). Other H atoms were placed in calculated positions with C—H = 0.93 and N—H = 0.86 Å, and refined in riding mode with *U*_iso_(H) = 1.2*U*_eq_(C,*N*).

### Crystal data

**Table:**

C_15_H_15_N_3_O	*F*_000_ = 536
*M**_r_* = 253.30	*D*_x_ = 1.301 Mg m^−^^3^
Monoclinic, *P*2_1_/*n*	Mo *K*α radiation λ = 0.71073 Å
Hall symbol: -P 2yn	Cell parameters from 3256 reflections
*a* = 12.261 (9) Å	θ = 2.0–25.0º
*b* = 5.324 (4) Å	µ = 0.08 mm^−^^1^
*c* = 19.882 (15) Å	*T* = 295 (2) K
β = 94.57 (2)º	Prism, yellow
*V* = 1293.7 (17) Å^3^	0.42 × 0.36 × 0.32 mm
*Z* = 4	

### Data collection

**Table:**

Rigaku R-AXIS RAPID IP diffractometer	2303 independent reflections
Radiation source: fine-focus sealed tube	1594 reflections with *I* > 2σ(*I*)
Monochromator: graphite	*R*_int_ = 0.042
Detector resolution: 10.00 pixels mm^-1^	θ_max_ = 25.2º
*T* = 295(2) K	θ_min_ = 1.9º
ω scans	*h* = −14→13
Absorption correction: none	*k* = −6→6
10914 measured reflections	*l* = −23→23

### Refinement

**Table:**

Refinement on *F*^2^	Hydrogen site location: inferred from neighbouring sites
Least-squares matrix: full	H-atom parameters constrained
*R*[*F*^2^ > 2σ(*F*^2^)] = 0.048	*w* = 1/[σ^2^(*F*_o_^2^) + (0.0957*P*)^2^] where *P* = (*F*_o_^2^ + 2*F*_c_^2^)/3
*wR*(*F*^2^) = 0.156	(Δ/σ)_max_ < 0.001
*S* = 1.06	Δρ_max_ = 0.21 e Å^−^^3^
2303 reflections	Δρ_min_ = −0.15 e Å^−^^3^
174 parameters	Extinction correction: SHELXL97 (Sheldrick, 2008), Fc^*^=kFc[1+0.001xFc^2^λ^3^/sin(2θ)]^-1/4^
Primary atom site location: structure-invariant direct methods	Extinction coefficient: 0.029 (5)
Secondary atom site location: difference Fourier map	

### Special details

**Table:**

Geometry. All e.s.d.'s (except the e.s.d. in the dihedral angle between two l.s. planes) are estimated using the full covariance matrix. The cell e.s.d.'s are taken into account individually in the estimation of e.s.d.'s in distances, angles and torsion angles; correlations between e.s.d.'s in cell parameters are only used when they are defined by crystal symmetry. An approximate (isotropic) treatment of cell e.s.d.'s is used for estimating e.s.d.'s involving l.s. planes.
Refinement. Refinement of *F*^2^ against ALL reflections. The weighted *R*-factor *wR* and goodness of fit *S* are based on *F*^2^, conventional *R*-factors *R* are based on *F*, with *F* set to zero for negative *F*^2^. The threshold expression of *F*^2^ > σ(*F*^2^) is used only for calculating *R*-factors(gt) *etc*. and is not relevant to the choice of reflections for refinement. *R*-factors based on *F*^2^ are statistically about twice as large as those based on *F*, and *R*- factors based on ALL data will be even larger.

### Fractional atomic coordinates and isotropic or equivalent isotropic displacement parameters (Å^2^)

**Table:**

	*x*	*y*	*z*	*U*_iso_*/*U*_eq_	
N1	0.31065 (12)	0.5389 (3)	0.53755 (7)	0.0458 (4)	
H1	0.3349	0.3881	0.5344	0.055*	
N2	0.21647 (12)	0.6131 (3)	0.49876 (7)	0.0445 (4)	
N3	−0.22800 (14)	0.7277 (3)	0.31088 (8)	0.0636 (5)	
H3A	−0.2549	0.6375	0.2777	0.076*	
H3B	−0.2616	0.8609	0.3223	0.076*	
O1	0.33735 (12)	0.9288 (3)	0.58300 (7)	0.0629 (5)	
C1	0.45840 (15)	0.6003 (3)	0.62353 (9)	0.0436 (5)	
C2	0.51973 (16)	0.3936 (4)	0.60532 (10)	0.0542 (6)	
H2	0.5019	0.3119	0.5646	0.065*	
C3	0.60765 (18)	0.3099 (4)	0.64827 (12)	0.0653 (6)	
H3	0.6489	0.1736	0.6357	0.078*	
C4	0.63412 (18)	0.4268 (4)	0.70911 (11)	0.0646 (6)	
H4	0.6927	0.3692	0.7375	0.078*	
C5	0.57340 (18)	0.6300 (4)	0.72783 (10)	0.0620 (6)	
H5	0.5904	0.7084	0.7691	0.074*	
C6	0.48743 (16)	0.7162 (4)	0.68502 (9)	0.0545 (6)	
H6	0.4479	0.8553	0.6976	0.065*	
C7	0.36407 (15)	0.7045 (4)	0.58023 (8)	0.0449 (5)	
C8	0.17939 (15)	0.4585 (3)	0.45231 (8)	0.0402 (5)	
C9	0.07430 (14)	0.5262 (3)	0.41483 (8)	0.0397 (5)	
C10	0.01535 (15)	0.7400 (3)	0.43293 (8)	0.0450 (5)	
H10	0.0437	0.8397	0.4685	0.054*	
C11	−0.08369 (16)	0.8054 (4)	0.39912 (9)	0.0480 (5)	
H11	−0.1199	0.9492	0.4118	0.058*	
C12	−0.12988 (15)	0.6578 (4)	0.34612 (9)	0.0477 (5)	
C13	−0.07350 (17)	0.4434 (4)	0.32890 (9)	0.0536 (6)	
H13	−0.1032	0.3403	0.2944	0.064*	
C14	0.02614 (16)	0.3814 (3)	0.36233 (9)	0.0496 (5)	
H14	0.0622	0.2378	0.3492	0.060*	
C15	0.23698 (17)	0.2176 (3)	0.43604 (10)	0.0544 (6)	
H15A	0.3141	0.2481	0.4361	0.082*	
H15B	0.2093	0.1587	0.3923	0.082*	
H15C	0.2241	0.0929	0.4694	0.082*	

### Atomic displacement parameters (Å^2^)

**Table:**

	*U*^11^	*U*^22^	*U*^33^	*U*^12^	*U*^13^	*U*^23^
N1	0.0451 (10)	0.0419 (9)	0.0490 (9)	0.0038 (7)	−0.0059 (7)	−0.0028 (7)
N2	0.0436 (10)	0.0446 (10)	0.0437 (8)	0.0004 (7)	−0.0059 (7)	0.0019 (7)
N3	0.0596 (12)	0.0707 (12)	0.0572 (10)	0.0061 (10)	−0.0166 (8)	0.0062 (9)
O1	0.0652 (10)	0.0461 (9)	0.0735 (10)	0.0083 (7)	−0.0176 (8)	−0.0082 (7)
C1	0.0390 (11)	0.0435 (11)	0.0478 (10)	−0.0025 (9)	0.0012 (8)	0.0016 (8)
C2	0.0469 (13)	0.0506 (12)	0.0643 (12)	0.0026 (9)	0.0000 (10)	−0.0059 (10)
C3	0.0582 (14)	0.0520 (13)	0.0851 (16)	0.0127 (11)	0.0029 (12)	0.0019 (11)
C4	0.0526 (14)	0.0656 (15)	0.0735 (15)	0.0092 (11)	−0.0078 (11)	0.0144 (12)
C5	0.0581 (14)	0.0714 (15)	0.0537 (12)	0.0061 (12)	−0.0128 (10)	−0.0033 (11)
C6	0.0530 (13)	0.0545 (13)	0.0545 (12)	0.0089 (10)	−0.0046 (9)	−0.0078 (10)
C7	0.0440 (12)	0.0439 (11)	0.0463 (10)	0.0004 (9)	0.0011 (8)	−0.0029 (8)
C8	0.0467 (11)	0.0343 (10)	0.0395 (9)	−0.0034 (8)	0.0020 (8)	0.0043 (7)
C9	0.0440 (11)	0.0346 (10)	0.0398 (9)	−0.0047 (8)	−0.0011 (8)	0.0047 (7)
C10	0.0487 (12)	0.0419 (11)	0.0433 (10)	−0.0021 (9)	−0.0031 (8)	−0.0043 (8)
C11	0.0474 (12)	0.0476 (12)	0.0487 (10)	0.0028 (9)	0.0030 (9)	0.0027 (9)
C12	0.0485 (12)	0.0492 (12)	0.0442 (10)	−0.0055 (9)	−0.0036 (9)	0.0126 (8)
C13	0.0646 (14)	0.0466 (12)	0.0462 (11)	−0.0057 (10)	−0.0160 (10)	0.0004 (9)
C14	0.0622 (14)	0.0380 (11)	0.0469 (10)	0.0019 (9)	−0.0071 (10)	−0.0020 (8)
C15	0.0584 (13)	0.0437 (12)	0.0588 (12)	0.0055 (10)	−0.0095 (10)	−0.0035 (9)

### Geometric parameters (Å, °)

**Table:**

N1—C7	1.355 (2)	C5—H5	0.9300
N1—N2	1.394 (2)	C6—H6	0.9300
N1—H1	0.8600	C8—C9	1.481 (3)
N2—C8	1.292 (2)	C8—C15	1.512 (3)
N3—C12	1.394 (2)	C9—C14	1.391 (2)
N3—H3A	0.8600	C9—C10	1.410 (3)
N3—H3B	0.8600	C10—C11	1.385 (3)
O1—C7	1.241 (2)	C10—H10	0.9300
C1—C6	1.390 (3)	C11—C12	1.398 (3)
C1—C2	1.396 (3)	C11—H11	0.9300
C1—C7	1.493 (3)	C12—C13	1.391 (3)
C2—C3	1.394 (3)	C13—C14	1.384 (3)
C2—H2	0.9300	C13—H13	0.9300
C3—C4	1.376 (3)	C14—H14	0.9300
C3—H3	0.9300	C15—H15A	0.9600
C4—C5	1.381 (3)	C15—H15B	0.9600
C4—H4	0.9300	C15—H15C	0.9600
C5—C6	1.380 (3)		
			
C7—N1—N2	120.00 (16)	N2—C8—C9	116.56 (16)
C7—N1—H1	120.0	N2—C8—C15	123.33 (17)
N2—N1—H1	120.0	C9—C8—C15	120.10 (16)
C8—N2—N1	116.36 (16)	C14—C9—C10	116.33 (17)
C12—N3—H3A	120.0	C14—C9—C8	122.89 (17)
C12—N3—H3B	120.0	C10—C9—C8	120.74 (16)
H3A—N3—H3B	120.0	C11—C10—C9	121.78 (17)
C6—C1—C2	118.19 (18)	C11—C10—H10	119.1
C6—C1—C7	118.33 (17)	C9—C10—H10	119.1
C2—C1—C7	123.48 (17)	C10—C11—C12	120.77 (18)
C3—C2—C1	119.9 (2)	C10—C11—H11	119.6
C3—C2—H2	120.0	C12—C11—H11	119.6
C1—C2—H2	120.0	C13—C12—N3	121.40 (18)
C4—C3—C2	120.7 (2)	C13—C12—C11	117.86 (18)
C4—C3—H3	119.6	N3—C12—C11	120.73 (19)
C2—C3—H3	119.6	C14—C13—C12	120.99 (17)
C3—C4—C5	119.8 (2)	C14—C13—H13	119.5
C3—C4—H4	120.1	C12—C13—H13	119.5
C5—C4—H4	120.1	C13—C14—C9	122.26 (18)
C6—C5—C4	119.8 (2)	C13—C14—H14	118.9
C6—C5—H5	120.1	C9—C14—H14	118.9
C4—C5—H5	120.1	C8—C15—H15A	109.5
C5—C6—C1	121.62 (19)	C8—C15—H15B	109.5
C5—C6—H6	119.2	H15A—C15—H15B	109.5
C1—C6—H6	119.2	C8—C15—H15C	109.5
O1—C7—N1	122.51 (17)	H15A—C15—H15C	109.5
O1—C7—C1	121.83 (16)	H15B—C15—H15C	109.5
N1—C7—C1	115.66 (17)		

### Hydrogen-bond geometry (Å, °)

**Table:**

*D*—H···*A*	*D*—H	H···*A*	*D*···*A*	*D*—H···*A*
N3—H3B···O1^i^	0.86	2.44	3.169 (3)	143
C15—H15C···N2^ii^	0.96	2.62	3.468 (3)	147

Symmetry codes: (i) −*x*, −*y*+2, −*z*+1; (ii) *x*, *y*−1, *z*.
